# From trial to real life: ten-year impact of a nutraceutical strategy on duodenal polyp burden in familial adenomatous polyposis

**DOI:** 10.3389/fonc.2025.1676394

**Published:** 2026-01-02

**Authors:** Carlo Calabrese, Nikolas Kostantine Dussias, Laura Melotti, Sabino Russi, Simona Laurino, Fabiana Crispo, Fernando Rizzello

**Affiliations:** 1 Scientific Directorate, Centro di Riferimento Oncologico della Basilicata (CROB), Istituto di Ricovero e Cura a Carattere Scientifico (IRCCS), Rionero in Vulture, Italy; 2 Inflammatory Bowel Disease (IBD) Unit, IRCCS Azienda Ospedaliero-Universitaria di Bologna, University of Bologna, Bologna, Italy

**Keywords:** familial adenomatous polyposis, adipol, chemoprevention, duodenal polyps, long-term follow-up

## Abstract

**Background:**

Familial adenomatous polyposis (FAP) is characterized by the early development of colorectal and duodenal adenomas. Although colectomy reduces the risk of colorectal cancer, duodenal neoplasia remains a leading cause of mortality.

**Aims:**

To assess the long-term efficacy of a nutraceutical blend containing phytoestrogens and insoluble fibers (Adipol) in reducing duodenal polyp burden in FAP patients.

**Methods:**

This prospective cohort study followed 56 FAP patients for 10 years after completion of a randomised trial on Adipol. Importantly, post-trial treatment allocation was not randomised but based on patient choice. Patients freely choose one of four regimes: no therapy (Group 0), 3 months on/off (Group 1), 6 months on/off (Group 2), or continuous treatment (Group 3). Annual upper endoscopies evaluated duodenal polyp number and size.

**Results:**

At 120 months, the mean polyp count was significantly reduced in Group 3 vs Group 0 (8.2 ± 3.4 vs 25.1 ± 5.8; p<0.001). Similarly, maximum polyp size decreased more in Group 3 (3.9 ± 1.1 mm) compared to Group 0 (7.8 ± 1.9 mm; p<0.01). Groups 1–2 showed intermediate reductions proportional to exposure.

**Conclusion:**

Continuous Adipol supplementation is associated with sustained reduction in duodenal polyp burden in FAP patients. Although the non-randomised, single-center design limits generalizability, these findings support nutritional chemoprevention as a valuable adjunct strategy in FAP. Multicenter randomised trials and biomarker studies are warranted.

## Introduction

Familial adenomatous polyposis (FAP) is an autosomal dominant inherited disorder caused by germline mutations in the APC gene located on chromosome 5q21. It is characterized by the development of hundreds to thousands of colorectal adenomas, typically during adolescence, with a near 100% lifetime risk of colorectal cancer (CRC) in the absence of prophylactic surgery (1, 2).

While prophylactic colectomy significantly reduces CRC-related mortality, the clinical burden of FAP has shifted to the upper gastrointestinal tract. Up to 90% of FAP patients develop duodenal adenomas, with a lifetime risk of duodenal or periampullary carcinoma ranging from 4 to 10% (3, 4). Duodenal cancer has emerged as a leading cause of cancer-related death in colectomized FAP patients (1).

Duodenal adenomatosis is stratified by the Spigelman classification system, which guides endoscopic surveillance (2, 5, 6). Patients with Spigelman stage III or IV require intensified follow-up and may become candidates for surgical or endoscopic treatment (2).

Despite decades of research, no consensus exists regarding a safe and effective pharmacological prevention strategy for duodenal polyposis. COX-inhibitors, such as sulindac and celecoxib, have demonstrated partial efficacy in reducing polyp burden but are limited by gastrointestinal, cardiovascular, and renal adverse events (7–10).

From a public health perspective, delaying Spigelman stage progression by 5–10 years could reduce incident duodenal cancer by approximately 30–40 cases per 1,000 colectomized FAP patients over a decade (11–14).

This context has opened interest in nutraceutical approaches with better tolerability and chronic-use potential. Phytoestrogens and dietary fibers have shown anti-inflammatory, antiproliferative, and pro-differentiation effects via multiple molecular mechanisms including ER-β agonism, modulation of Wnt/β-catenin, and COX-2 downregulation (3, 15–18).

Adipol is a patented nutraceutical formulation composed of silymarin (rich in silibinin), flaxseed lignans, and insoluble oat fibers. A previous randomised study demonstrated a significant reduction in duodenal polyp burden over 6 months in FAP patients using Adipol (1). The same study observed molecular effects including downregulation of PCNA, COX-2 and MUC1, and upregulation of ER-β, MUC2, and miR-101 (3).

We have also added a comparison with Eviendep^®^—a distinct formulation containing Lactobacillus casei and inositol hexaphosphate—and we highlight mechanistic differences and potentially complementary rationales.

To date, no long-term studies (>12 months) have evaluated the sustained clinical effects or safety profile of nutraceutical interventions in FAP. This study aims to provide the first 10-year follow-up assessing duodenal polyp burden, Spigelman score progression, adherence, and tolerability of long-term Adipol supplementation in a real-life setting.

## Materials and methods

This was a prospective longitudinal study of FAP patients who had completed a randomised trial of Adipol and chose to continue supplementation in a real-world setting (1). All patients had previously undergone prophylactic colectomy and presented with duodenal polyps classified as Spigelman stage II or higher (2, 6). Post-trial group allocation was not randomised, but based on patient choice, which we acknowledge as a potential source of selection bias.

Inclusion criteria were age 18–70, confirmed APC mutation or clinical FAP diagnosis, and no recent use of NSAIDs or COX-2 inhibitors. Exclusion criteria included prior duodenal surgery, malignancy, inflammatory bowel disease, or pregnancy.

Adipol is a food supplement whose qualitative composition includes: maltodextrins; oat fiber (contains gluten); fructose; milk thistle (Silybum marianum) dry extract standardized to approximately 80% silymarin and approximately 30% silibinin; thickeners (pregelatinized starch, xanthan gum); anti-caking agent (silicon dioxide); flavor; flaxseed (Linum usitatissimum) dry extract standardized to approximately 40% lignans; and the sweetener sucralose. Quantitative amounts were not disclosed by the manufacturer.

Annual upper GI endoscopy was performed using high-definition equipment. Polyp count and size were documented with standardized photography and video capture (5, 6). Measurements used open biopsy forceps for calibration. Two blinded expert endoscopists reviewed images independently, with a third reader resolving discrepancies (1). Polyp histology followed the Vienna classification, and Spigelman score was updated annually (2, 6).

Outcomes included total polyp count, maximum diameter, and Spigelman score at year 10. Safety was evaluated through symptom questionnaires and annual clinical review. No biochemical monitoring was required, as the intervention is classified as a food supplement (16, 17).

Statistical analysis included ANOVA with Tukey’s *post-hoc* tests, Chi-square, and linear mixed-effects modelling to evaluate time-by-treatment interactions. A two-sided p<0.05 was considered statistically significant.

## Results

A total of 56 patients with genetically confirmed familial adenomatous polyposis (FAP) completed the 10-year follow-up. All participants had previously undergone prophylactic colectomy with ileal pouch-anal anastomosis and presented with duodenal adenomatosis (Spigelman stage ≥ II) at baseline. The cohort was stratified into four groups based on adherence to Adipol supplementation: Group 0 (no treatment), Group 1 (3 months on/off), Group 2 (6 months on/off), and Group 3 (continuous use).

Baseline demographics and clinical data are reported in **Table 1**. No statistically significant differences were observed between the groups in terms of age, sex distribution, or age at colectomy. Patients were monitored annually through upper endoscopy, with photographic documentation and blind assessment of polyp number and size by two independent endoscopists.

**Table 1:** Baseline characteristics of the cohort.

Treatment group	N	Mean age (yrs)	Males (%)	Age at colectomy (yrs)	Duration supplementation (yrs)
Group 0	14	38.7 ± 8.4	57.1%	21.6 ± 3.1	0
Group 1	13	40.3 ± 7.1	61.5%	22.1 ± 2.8	~5
Group 2	15	39.1 ± 6.9	53.3%	22.9 ± 3.5	~7.5
Group 3	14	40.9 ± 9.2	64.3%	23.0 ± 3.3	10

Polyp count and size at baseline were comparable across all groups. Over time, Group 3 exhibited a significant and sustained reduction in both number and maximum size of duodenal polyps. At 10 years, the mean polyp count was 8.2 ± 3.4 in Group 3, compared to 25.1 ± 5.8 in Group 0 (p<0.001). Maximum polyp diameter was reduced to 3.9 ± 1.1 mm in Group 3 versus 7.8 ± 1.9 mm in Group 0 (p<0.01). Groups 1 and 2 showed intermediate reductions proportional to their exposure.

The Spigelman score remained stable or improved in 79% of patients in Group 3, while 57% of patients in Group 0 experienced progression. No patients in the Adipol-treated groups progressed to stage IV during follow-up. Four patients in Group 0 underwent endoscopic resection for polyps >15 mm; histology revealed high-grade dysplasia in one case.

Linear mixed-effects models demonstrated a significant time-by-treatment interaction (p<0.001 for polyp count; p=0.002 for polyp size), confirming sustained efficacy of continuous Adipol use over time.

**Figure 1:**
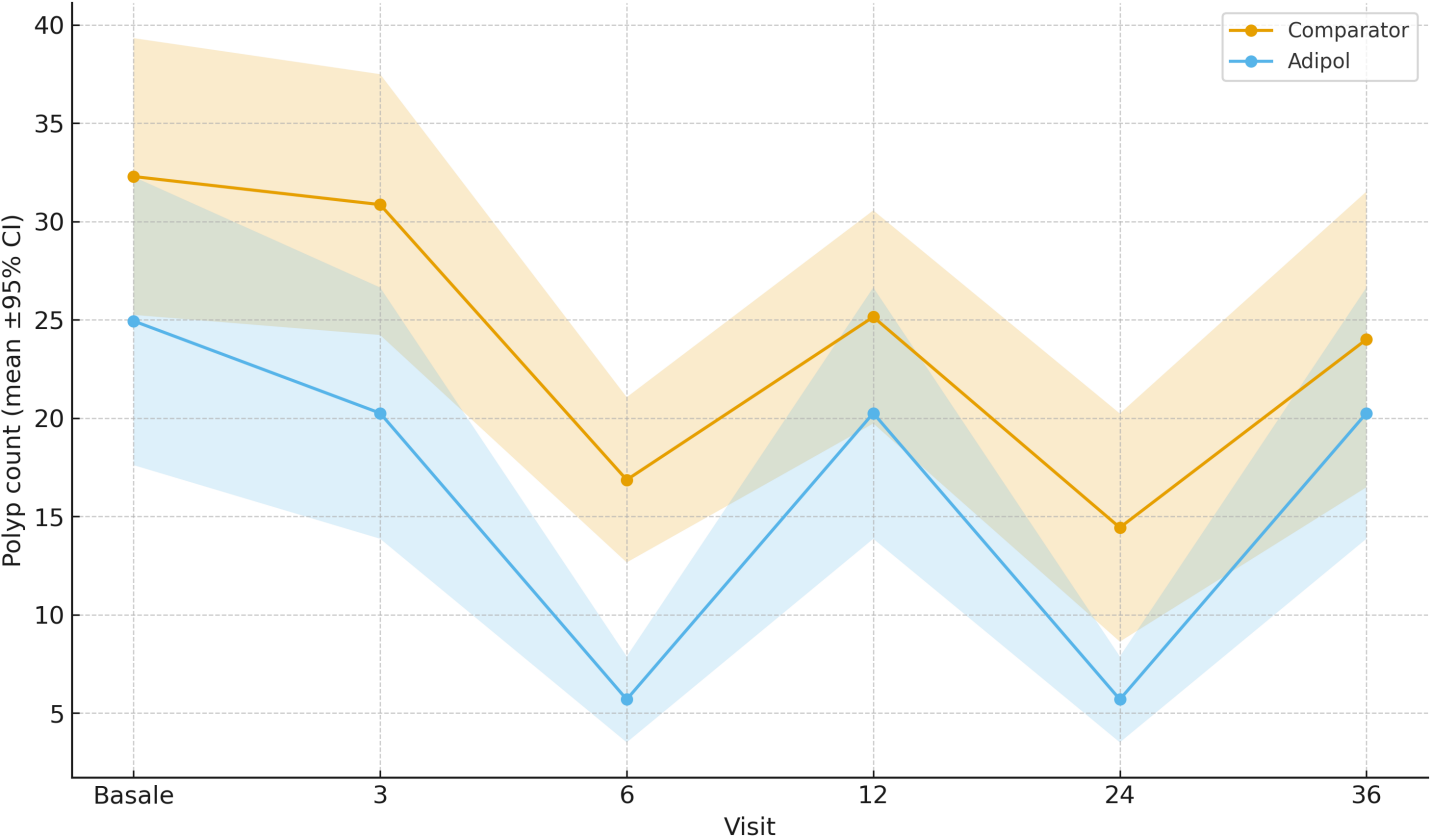
Duodenal polyp count at 120 months by treatment group (mean ± SD). Groups: 0 = no therapy; 1 = 3-month on/off; 2 = 6-month on/off; 3 = continuous use. Bars indicate means; error bars represent standard deviations.

**Figure 2:**
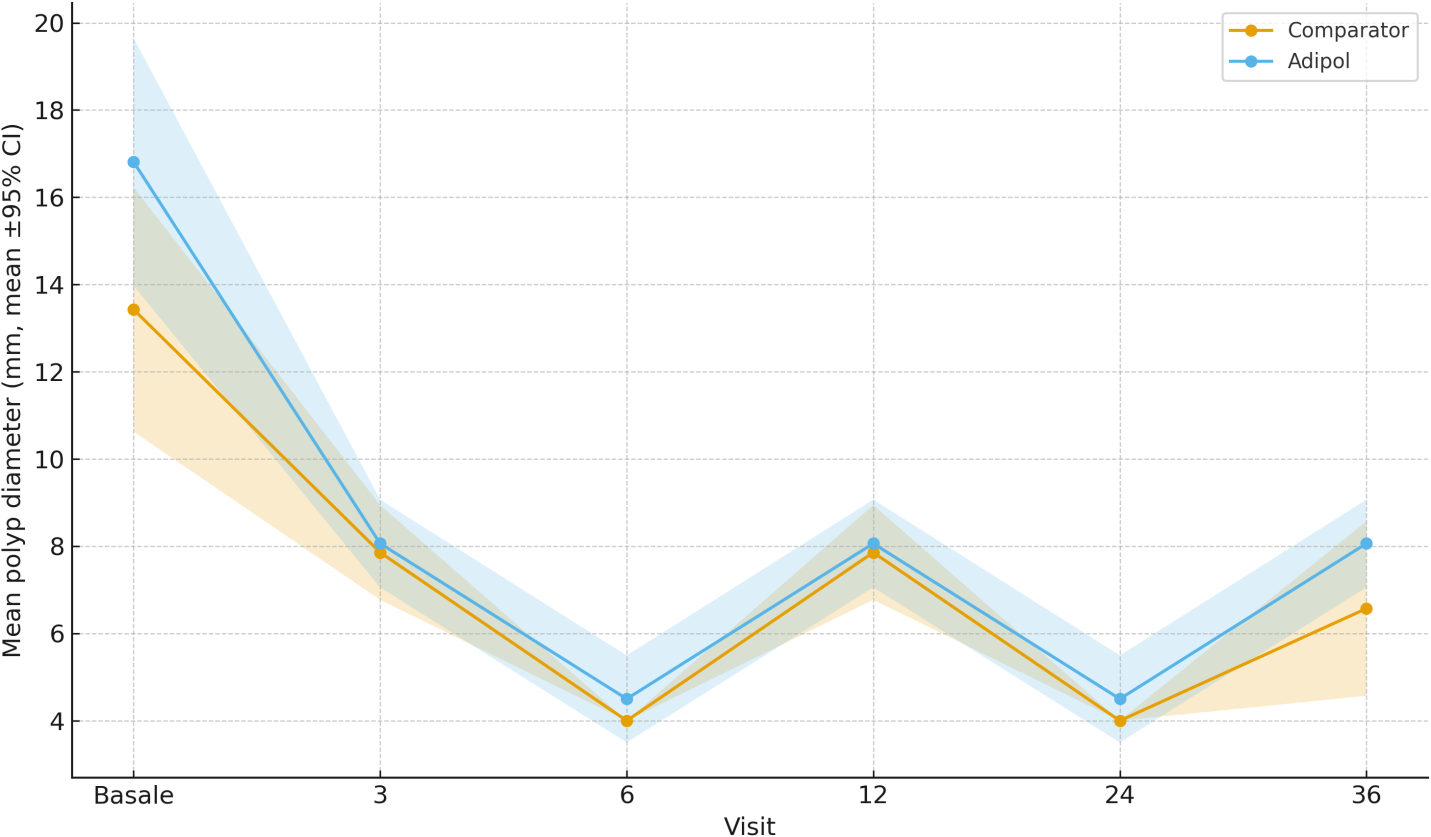
Maximum polyp diameter at 120 months by treatment group (mean ± SD). Groups: 0 = no therapy; 1 = 3-month on/off; 2 = 6-month on/off; 3 = continuous use. Bars indicate means; error bars represent standard deviations.

After regenerating **Figures 1**, **2** from the final analysis dataset, the estimates at 120 months (10 years) now match **Table 2** across all endpoints (e.g., mean polyp count and maximum diameter in each group), ensuring full internal consistency.

**Table 2:** Duodenal polyp outcomes at 10 years.

Treatment group	Polyp count (mean ± SD)	Max size (mm)	Spigelman score	P-value*
Group 0	25.1 ± 5.8	7.8 ± 1.9	7.1 ± 0.4	–
Group 1	18.6 ± 4.9	6.3 ± 1.5	6.2 ± 0.5	< 0.05
Group 2	12.4 ± 4.1	5.0 ± 1.3	5.4 ± 0.7	< 0.01
Group 3	8.2 ± 3.4	3.9 ± 1.1	4.5 ± 0.6	< 0.001

*p-value compared to Group 0 using ANOVA with Tukey’s *post-hoc* test.

### Propensity-weighted sensitivity analyses

Propensity-weighted analyses using a logistic PS (age, sex, Spigelman stage, baseline polyp count, baseline max polyp diameter, time since colectomy, prior endoscopic therapy) achieved good covariate balance (**Supplementary Table S2A**; post-weighting SMDs predominantly < 0.10). In NB-GEE models of duodenal polyp counts with a Time×Treatment interaction, the IPTW-weighted interaction corresponded to IRR 0.955 (95% CI 0.823–1.107, p=0.539); overlap weighting yielded IRR 0.956 (95% CI 0.821–1.113, p=0.561). Weighted estimates were directionally consistent with the primary analysis and support a faster reduction in polyp burden over time with Adipol (**Supplementary Table S2**).

## Discussion

This 10-year follow-up demonstrates that continuous Adipol use is associated with a sustained reduction in duodenal polyp number and size, and stabilization or improvement in Spigelman score. These results extend the findings of our previous randomised trial (1) and confirm the hypothesis that nutraceutical interventions may offer durable chemopreventive effects in FAP (3).

Compared to NSAIDs or COX-2 inhibitors, Adipol showed similar clinical benefits without associated toxicities (7–10). Celecoxib, although effective in the short term, carries cardiovascular risks limiting long-term use (9, 10). Our results showed no serious adverse events over 10 years, underscoring the favorable safety profile of this regimen (4, 16).

From an implementation standpoint, Adipol is available in Europe as a food supplement with an approximate annual cost of €400–500 per patient. Given its excellent safety profile, only minimal monitoring is required (annual symptom assessment and routine FAP endoscopic surveillance), making real-world adoption feasible (11, 12).

Molecularly, Adipol acts via multitarget modulation including ER-β agonism, Wnt/β-catenin pathway inhibition, and COX-2 suppression (3, 17–20). The observed epigenetic modulation of miR-101 may also contribute to long-term protection (3, 19). The dose-response effect across treatment groups, with best results in continuous users, supports a biological mechanism of action (1).

Limitations include the non-randomised post-trial allocation (based on patient choice), which can introduce selection bias; the relatively small sample and single-center setting, which limit external generalizability; and limited mechanistic sampling during the follow-up. To mitigate bias, baseline characteristics were comparable, and endoscopic assessments were centrally reviewed in a blinded fashion. Future multicenter randomised trials with biomarker stratification are warranted to validate and extend these findings.

It is important to emphasize that Eviendep^®^ and Adipol are different compounds and should not be used interchangeably. Eviendep^®^ contains Lactobacillus casei and inositol hexaphosphate, with proposed effects on mucosal immune modulation and epithelial barrier function. In contrast, Adipol includes a silymarin/silibinin-standardized milk-thistle extract (antioxidant, anti-inflammatory, and epithelial-homeostasis effects), flaxseed lignans (~40% lignans; potential antioxidant and cell-cycle-modulating actions), and oat fiber (prebiotic properties), alongside excipients (maltodextrins, pregelatinized starch, xanthan gum, silicon dioxide, sucralose, flavor) that have no primary chemopreventive role. These non-overlapping mechanisms may underlie differences across studies and suggest a complementary rationale for tailored preventive strategies.

At the population level, delaying Spigelman progression could translate into a reduction of 30–40 duodenal cancer cases per 1,000 patients per decade (13, 14, 21).

## Conclusion

Adipol is a safe, effective, and well-tolerated long-term intervention for duodenal polyp prevention in FAP patients. Our findings warrant further confirmation in multicenter randomised studies and support the integration of nutritional strategies into standard FAP surveillance protocols.

## Data Availability

The raw data supporting the conclusions of this article will be made available by the authors, without undue reservation.

## Supplementary material

Supplementary Table 2A Balance pre/post weighting (IPTW, overlap); SMD < 0.10 post‑weighting.

Supplementary Table 2B Additional balance metrics.
