# Supplementary material for: From trial to real life: ten-year impact of a nutraceutical strategy on duodenal polyp burden in familial adenomatous polyposis
Source: Front Oncol. 2026 Jan 2;15:1676394. doi: 10.3389/fonc.2025.1676394 (PMC12807981; doi:10.3389/fonc.2025.1676394)
Supplement: Supplementary Table 2A — Balance pre/post weighting (IPTW, overlap); SMD < 0.10 post‑weighting. [file SupplementaryFile1.pdf]

Table S1. Safety summary over 10 years (AE/SAE)

System Organ Class (SOC)	Preferred Term	Severity (CTCAE grade)	Relatedness	Serious (Y/N)	n events	n patients (%)	Outcome	Notes
Overall	—	—	No product-related AEs/SAEs observed	N	0	0 (0%)	—	Ten-year follow-up; detailed listing pending.

Footnotes: AE = Adverse Event; SAE = Serious Adverse Event; Severity per CTCAE v5.0.

Table S2A. Covariate balance before and after weighting (IPTW, overlap weighting)

Covariate	Unweighted SMD	SMD after IPTW	SMD after Overlap	Balance OK (<0.10)
Baseline polyp count	-0.195			No
Baseline mean polyp diameter (mm)	0.502			No

Abbreviations: SMD = standardized mean difference; IPTW = inverse probability of treatment weighting. Target: SMD < 0.10.

Table S2B. Model estimates (current unweighted; PS-weighted pending covariates)

Outcome	Model	Weighting method	Effect (IRR or β)	95% CI	p-value	Interpretation (concise)
Polyp count (Time×Treatment, per 1 year)	NB-GEE	Unweighted	IRR 1.044	0.934–1.167	0.446	Interaction IRR < 1 indicates faster reduction with Adipol over time.
Mean polyp diameter (Time×Treatment, per 1 year)	Linear mixed-effects	Unweighted	β -0.233	-1.267–0.801	0.661	Interaction not statistically significant.
